# Role of HMGB1 in Chemotherapy-Induced Peripheral Neuropathy

**DOI:** 10.3390/ijms22010367

**Published:** 2020-12-31

**Authors:** Fumiko Sekiguchi, Atsufumi Kawabata

**Affiliations:** Laboratory of Pharmacology and Pathophysiology, Faculty of Pharmacy, Kindai University, 3-4-1 Kowakae, Higashi-Osaka 577-8502, Japan; fumiko@phar.kindai.ac.jp

**Keywords:** high mobility group box 1 (HMGB1), chemotherapy-induced peripheral neuropathy (CIPN), thrombomodulin alfa (TMα)

## Abstract

Chemotherapy-induced peripheral neuropathy (CIPN), one of major dose-limiting side effects of first-line chemotherapeutic agents such as paclitaxel, oxaliplatin, vincristine, and bortezomib is resistant to most of existing medicines. The molecular mechanisms of CIPN have not been fully understood. High mobility group box 1 (HMGB1), a nuclear protein, is a damage-associated molecular pattern protein now considered to function as a pro-nociceptive mediator once released to the extracellular space. Most interestingly, HMGB1 plays a key role in the development of CIPN. Soluble thrombomodulin (TMα), known to degrade HMGB1 in a thrombin-dependent manner, prevents CIPN in rodents treated with paclitaxel, oxaliplatin, or vincristine and in patients with colorectal cancer undergoing oxaliplatin-based chemotherapy. In this review, we describe the role of HMGB1 and its upstream/downstream mechanisms in the development of CIPN and show drug candidates that inhibit the HMGB1 pathway, possibly useful for prevention of CIPN.

## 1. Introduction

Chemotherapy-induced peripheral neuropathy (CIPN) is one of the major dose-limiting side effects of first-line chemotherapeutic agents such as paclitaxel, oxaliplatin, vincristine, and bortezomib. In terms of evidence-based medicine, no drugs are recommended for the prevention of CIPN, and duloxetine is the only agent that has limited benefit in treating established CIPN [1]. The mechanisms for development and maintenance of CIPN have not been fully understood, although preclinical studies have provided evidence for some possible mechanisms for CIPN [2], such as neuroimmune interactions [3], mitochondrial dysfunction [4], reactive oxygen species (ROS) accumulation [5], and transcriptional or functional upregulation of cation channels [6,7] in the spinal cord, dorsal root ganglion (DRG), and peripheral sensory neurons. Our preclinical studies have demonstrated the involvement of high mobility group box 1 (HMGB1) in the development and maintenance of CIPN [8,9,10,11]. HMGB1 (also known as amphoterin) is a non-histone nuclear protein that has highly conserved amino acid sequences during evolution and is ubiquitously expressed in most mammalian cell types [12]. HMGB1 is essential for life, because HMGB1-deficient mice die within 24 h of birth due to hypoglycemia [13]. In the nucleus, HMGB1 contributes to nucleosome stability and sliding, DNA replication and repair, gene transcription, etc. On the other hand, HMGB1 is actively secreted by activated immune cells and passively released from necrotic cells in pathological conditions including inflammation. Extracellular HMGB1 acts as a damage-associated molecular pattern (DAMP) protein via activation of several pattern recognition receptors (PRRs) including Toll-like receptors (TLRs), receptors for advance glycosylation end products (RAGE), C-X-C motif chemokine receptor 4 (CXCR4), etc., leading to acceleration of inflammation and pain [11,12,14,15,16,17,18,19,20]. There are plenty of papers showing that an anti-HMGB1-neutralizing antibody (HMGB1-nAb) [21,22] strongly suppresses somatic or visceral pathological pain with inflammatory and/or neuropathic components [23,24,25,26,27,28,29,30,31,32,33,34]. We have shown that the HMGB1-nAb strongly prevents the development of CIPN in rodents [8,9,10]. Similarly, recombinant human soluble thrombomodulin (TM) [thrombomodulin alfa (TMα), ART-123, recomodulin^®^], capable of promoting thrombin-dependent HMGB1 degradation [11,35,36], also prevents the development of CIPN in rodent models [8,9,10]. Intriguingly, the efficacy of TMα in preventing CIPN in humans has been confirmed by a placebo-controlled, randomized, double-blind phase IIa study [37]. Thus, targeting extracellular HMGB1 is considered a promising strategy to prevent CIPN. In this review, we focus on the role of HMGB1 in the development of CIPN, and describe large or small molecules that deactivate HMGB1 or inhibit the upstream/downstream signaling molecules of HMGB1, which may be useful for prevention of CIPN.

## 2. Molecular and Biological Characteristics of HMGB1

### 2.1. Structure of HMGB1

**Figure 1:**
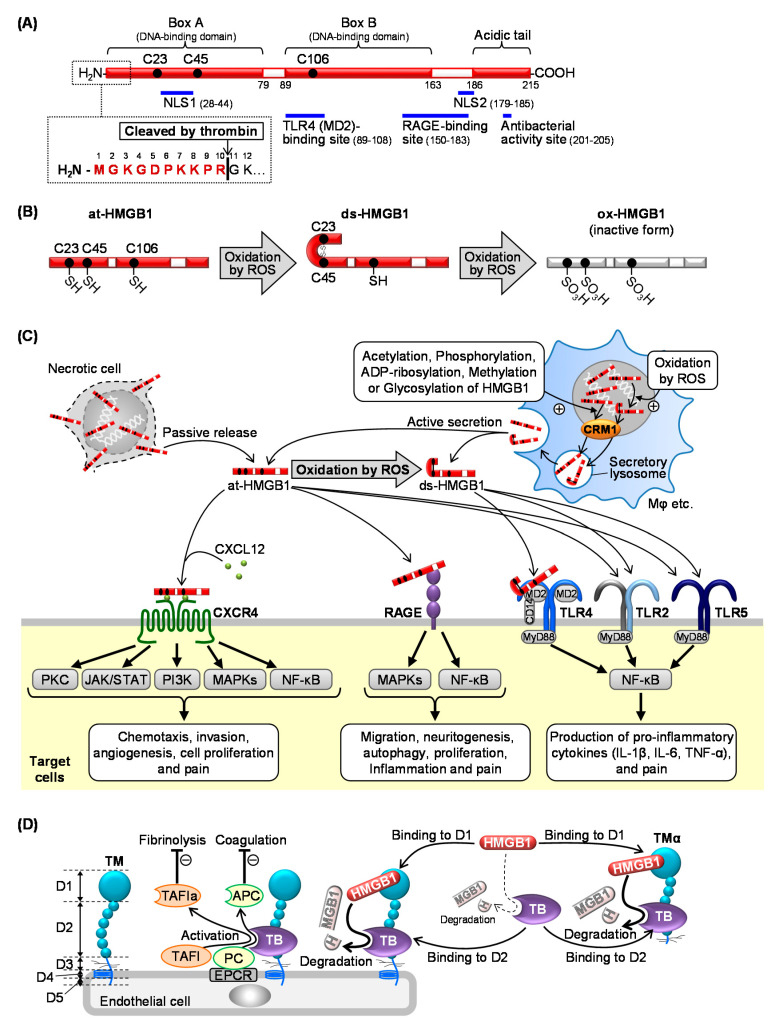
Structure, origins/targets, and inactivation of high mobility group box 1 (HMGB1). (**A**) The structure and thrombin cleavage site of HMGB1. (**B**) Redox-dependent structural conformation of HMGB1. (**C**) Extracellular release and membrane receptors of HMGB1. (**D**) Thrombin-dependent degradation of HMGB1 by thrombomodulin and thrombomodulin alfa. NLS, nuclear-localization signal; at-HMGB1, all-thiol HMGB1; ds-HMGB1, disulfide HMGB1; ox-HMGB1, fully-oxidized HMGB1; ROS, reactive oxygen species; CRM1, chromosome-region maintenance 1; TB, thrombin; ROS, reactive oxygen species; CXCR4, C-X-C motif chemokine receptor 4; CXCL12, C-X-C motif chemokine ligand 12; RAGE, receptor for advance glycosylation end products; TLR, Toll-like receptor; TM, thrombomodulin; TMα, thrombomodulin alfa; TAFI, thrombin-activatable fibrinolysis inhibitor; TAFIa, the activated form of TAFI; PC, protein C; APC, activated protein C; D1, D2, D3, D4, and D5, domain 1, 2, 3, 4, and 5 of thrombomodulin; EPCR, endothelial protein C receptor.

HMGB1 consists of 215 amino acids, the sequences of which are highly conserved among different mammalian species, e.g., 99% identical between rodents and humans [12]. HMGB1 has two positively charged DNA-binding domains (box A and box B), which are necessary for efficient DNA bending and flexure without sequence specificity, and a negatively charged C-terminal acidic tail, including a domain responsible for the antibacterial activity of HMGB1 (amino acids 201–205) (Figure 1A) [12,38]. Two nuclear-localization signals, NLS1 (amino acids 28–44) and NLS2 (amino acids 179–185), under the steady state, are responsible for localization of HMGB1 in the nucleus (Figure 1A) [39]. Two regions, amino acids 150–183 and 89–108, are responsible for binding to RAGE and TLR4, respectively (Figure 1A) [40,41]. HMGB1 has three cysteine residues (C23, C45, and C106) the redox forms of which are associated with the biological activity of HMGB1: (1) a fully reduced form, “all-thiol HMGB1 (at-HMGB1)” has three cysteine residues in a thiol form; (2) a partially oxidized form, “disulfide HMGB1 (ds-HMGB1)” has a disulfide bridge between C23 and C45; (3) “fully-oxidized HMGB1 (ox-HMGB1)” has three cysteine residues in a sulfhydryl form (Figure 1B) [42]. Biologically, at-HMGB1 and ds-HMGB1, but not ox-HMGB1, are active and play separate physiological and/or pathological roles [43].

### 2.2. Release of HMGB1

HMGB1 is passively released from necrotic cells and can also be actively secreted by immune cells (Figure 1C) such as macrophages [44], microglia [45], neutrophils [46], and natural killer cells [47], and also by non-immune cells, such as fibroblasts [48], epithelial cells [49,50], neurons [33], platelets [51], hepatocytes [52], and cardiomyocytes [53]. The active secretion of HMGB1 is triggered by microbial pathogens such as lipopolysaccharide (LPS) and polyinosinic-polycytidylic acid (poly(I:C)) [44,52,54], or endogenous substances, such as ROS [55], reactive nitrogen species (RNS) [56], hyperglycemia [53], inflammatory cytokines (e.g., TNF-α [49], interferon (IFN)-α [54], INF-γ [57], ATP [19,58], nitric oxide [54], calcium phosphate-based mineralo-organic particles [46], and also by cell-to-cell interaction [47]. Cytoplasmic translocation of nuclear HMGB1 is triggered and/or modulated by post-translational molecular modifications of HMGB1, such as acetylation [39,59], phosphorylation [60], ADP-ribosylation [61], methylation [62], glycosylation [63], and ROS-induced oxidation [64] (Figure 1C). The interaction between HMGB1 and chromosome-region maintenance 1 (CRM1), also known as exportin-1, plays a key role in the cytoplasmic translocation of nuclear HMGB1. The cytoplasmic HMGB1 is packaged in secretory lysosomes and then released to the extracellular space (Figure 1C), while the classical endoplasmic reticulum-Golgi secretory pathway is not involved in the HMGB1 secretion [64,65].

### 2.3. Membrane Receptors of Extracellular HMGB1 Involved in Pain Processing

Extracellular HMGB1 triggers or accelerates activation of various membrane receptors including RAGE, TLRs (TLR2, TLR4, TLR5), CD24, NMDA receptor, TIM-3, haptoglobin, and CXCR4 [12,14,66]. Among these receptors, RAGE, TLRs, and CXCR4 appear to mainly mediate the HMGB1-dependent pain signaling (Figure 1C) [11,15,66].

#### 2.3.1. RAGE

RAGE is a 45-kDa transmembrane receptor that belongs to the immunoglobulin superfamily, and widely expressed in various types of cells, such as immune cells (macrophages, neutrophils, and mast cells), endothelial cells, and neurons [67]. Accumulating evidence reveals that RAGE contributes to the pathogenesis of many diseases, including diabetic complications, Alzheimer′s disease, cardiovascular diseases, arthritis, and cancer [15,67,68,69]. It is of note that RAGE is overexpressed in the vast majority of cancer cells [68]. RAGE was originally identified as a receptor for advanced glycation end products (AGEs), but is now known to respond to at-HMGB1, S100 proteins, and amyloid β [15]. RAGE, when stimulated, activates cell signals including mitogen-activated protein kinases (MAPKs) and nuclear factor-κB (NF-κB) (Figure 1C) [68,69]. A number of studies demonstrate that RAGE is required for HMGB1-induced inflammation [70], pain [16,19,30,32], cell migration [71], neuritogenesis [72], autophagy [73], and proliferation [74,75]. We have demonstrated that RAGE mediates the allodynia/hyperalgesia following intraplantar administration of at-HMGB1, but not ds-HMGB1 [16], and that the HMGB1/RAGE pathway plays a crucial role in the neuropathic pain caused by spinal nerve injury [76] and visceral pain accompanying cystitis [19,28] and pancreatitis [32] (Figure 1C).

#### 2.3.2. TLRs

TLRs are an evolutionarily-conserved type I transmembrane superfamily that contain extracellular leucine-rich repeat domains, and have ten members in humans including cell surface receptors (TLR2, TLR4, TLR5, TLR6, and TLR10) and receptors in intracellular compartments (TLR3, TLR7, TLR8, and TLR9) [12,15,77]. TLRs are expressed in innate immune cells (dendritic cells and macrophages) as well as non-immune cells including fibroblasts and epithelial cells [77]. TLRs recognize pathogen-associated molecular pattern (PAMP) and DAMP molecules, followed by activation of the MyD88-dependent NF-κB pathway for production of pro-inflammatory cytokines (IL-1β, IL-6, and TNF-α) and the MyD88-independent IRF pathway for production of interferon [12,77]. Extracellular HMGB1 interacts with membrane receptors including TLR2, a receptor for lipopeptide, TLR4, a receptor for LPS, and TLR5, a receptor for flagellin, leading to the development or aggravation of inflammatory and pain signals (Figure 1C) [16,78,79,80]. HMGB1 binding to nucleic acids penetrates into the cells, and stimulates endosomal TLR9, which is responsible for innate immunity and autoimmunity [12,15,81,82]. The molecular mechanisms for TLR4 activation by HMGB1 have been well studied; i.e., ds-HMGB1, but not at-HMGB1, stimulates TLR4 by binding to MD-2 [12,43]. Among TLR family members, TLR4 and TLR5 mediate HMGB1-induced pain signals (Figure 1C) [11,78]. Interestingly, there is evidence that HMGB1 binding to RAGE promotes translocation of TLR4 to the cell surface, and that HMGB1 binding to TLR4 promotes expression of RAGE [70].

#### 2.3.3. CXCR4

CXCR4, a G protein-coupled receptor activated by chemokine C-X-C motif ligand 12 (CXCL12), also known as stromal cell-derived factor-1 (SDF-1), is widely expressed in various cells including endothelial/epithelial cells, fibroblasts, and neurons [83,84]. CXCR4 is strongly expressed in various types of cancer cells, contributing to tumorigenesis and cancer progression, e.g., chemotaxis, invasion, angiogenesis, and cell proliferation [83]. The CXCL12/CXCR4 axis is also involved in pathological pain, particularly neuropathic pain [85]. HMGB1, possibly at-HMGB1, is capable of accelerating the CXCL12/CXCR4 signaling (Figure 1C) [86]. Each of two CXCL12 molecules binds to box A and box B in an HMGB1 molecule [86,87], forming a heterocomplex which in turn causes dimerization of CXCR4 and consequently leads to greater responses than the conventional CXCL12/CXCR4-mediated signaling (Figure 1C) [86,87].

### 2.4. Inactivation of HMGB1 by the Thrombin and TM System

Thrombin at high concentrations (5 U/mL or higher) inactivates HMGB1 by cleaving a peptide bond between Arg10 and Gly11 of HMGB1 (Figure 1A). Endothelial TM, known to accelerate proteolytic activation of protein C (PC) and thrombin-activatable fibrinolysis inhibitor (TAFI) by thrombin, causes HMGB1 degradation in the presence of thrombin at low or minimal concentrations (2 U/mL or lower), an effect mimicked by TMα, a recombinant human soluble TM. TM, as well as TMα, sequesters HMGB1 and accelerates thrombin-induced HMGB1 degradation (Figure 1D) [11,35,36,88,89]. The endothelial TM/thrombin axis may thus play a role in decomposing excessive HMGB1 in the blood stream under pathological conditions [11].

## 3. Role of HMGB1 in CIPN

### 3.1. Involvement of Endogenous HMGB1 in the Development of Pathological Pain Including CIPN

**Figure 2:**
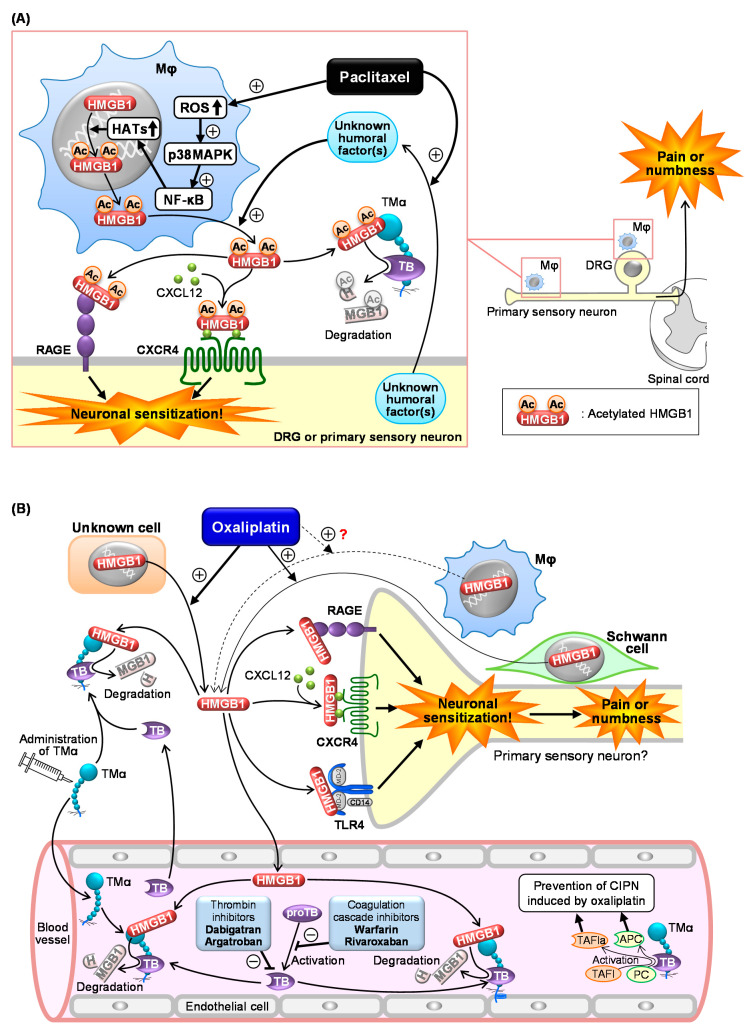
Scheme of the mechanisms underlying chemotherapy-induced peripheral neuropathy (CIPN) in mice treated with paclitaxel (**A**) or oxaliplatin (**B**). (**A**) Paclitaxel causes macrophage (Mφ) accumulation in the dorsal root ganglion (DRG) and sciatic nerves (primary sensory neurons) and upregulates histone acetyltransferases (HATs) via the ROS/p38MAPK/NF-κB pathway, resulting in acetylation and cytoplasmic translocation of nuclear HMGB1 followed by its secretion. Unknown humoral factors released from neurons in response to paclitaxel promote the HMGB1 release from Mφ. The secreted HMGB1 causes neuronal excitation through activation of RAGE, TLR4, and the CXCL12/CXCR4 axis, leading to CIPN characterized by pain or numbness. TMα prevents CIPN following paclitaxel treatment by degrading extracellular HMGB1 in a thrombin (TB)-dependent manner. (**B**) Oxaliplatin induces HMGB1 release possibly from multiple cells including Schwann cells, leading to CIPN through neuronal sensitization via activation of RAGE, TLR4, and CXCL12/CXCR4 pathways. Exogenously applied TMα and endothelial TM accelerate thrombin-induced degradation of HMGB1, thereby reducing the CIPN induced by oxaliplatin. Anticoagulants such as dabigatran, argatroban, warfarin, and rivaroxaban reduce the thrombin-dependent degradation of HMGB1 by TM and TMα, thereby aggravating CIPN and cancelling the anti-CIPN effect of TMα.

In 2001, it was reported for the first time that injection of HMGB1 around the sciatic nerve induced mechanical allodynia in rats [90]. Recently, increasing evidence has unveiled the pro-nociceptive role of HMGB1 in the peripheral tissue and spinal cord [16,18,29,80,89], and demonstrated that endogenous HMGB1 is involved in the pathogenesis of various types of intractable pain [11,15], including inflammatory pain [28,29,80], visceral pain [19,20,30,32], neuropathic pain [23,31,76,91,92], cancer pain [33], and post-stroke pain [34]. Endogenous HMGB1 also appears to play a key role in the development of CIPN in rats or mice treated with cancer chemotherapeutics, such as paclitaxel, oxaliplatin, and vincristine, considering the complete prevention of CIPN by inactivation of HMGB1 with HMGB1-nAb or TMα (Figure 2 and Table 1) [8,9,10,11].

### 3.2. Roles of Macrophage-Derived HMGB1 and Its Upstream/Downstream Molecules in CIPN Caused by Paclitaxel

Macrophages appear to be the main source of HMGB1 involved in CIPN caused by paclitaxel (Figure 2A) [9]. Depletion of macrophages by liposomal clodronate or treatment with minocycline and ethyl pyruvate, capable of inhibiting HMGB1 release from macrophages, completely abolishes the development of CIPN in mice treated with paclitaxel (Table 1) [9]. Actually, macrophages accumulate in the sciatic nerve and dorsal root ganglion (DRG) from the CIPN mice treated with paclitaxel (Figure 2A) [9,93]. In macrophage-like RAW264.7 cells, paclitaxel causes cytoplasmic translocation and extracellular secretion of nuclear HMGB1, which involves the activation of the ROS/p38MAPK/NF-κB pathway and subsequent upregulation of histone acetyltransferases (HATs), possibly essential for acetylation of HMGB1 (Figure 2A) [9]. Deletion of ROS by *N*-acetylcysteine (NAC), an antioxidant, prevents the development of CIPN in mice treated with paclitaxel in vivo (Table 1), in agreement with the in vitro experiments using macrophages [9]. Co-culture experiments using macrophage-like RAW264.7 cells and neuron-like NG108-15 cells suggest that “unknown humoral factors” derived from neurons accelerate HMGB1 secretion by macrophages in response to paclitaxel (Figure 2A) [9]. Thus, a crosstalk between macrophages and neurons mediated by HMGB1 and unknown humoral factors may play a key role in CIPN development after paclitaxel treatment.

Antagonists of RAGE or CXCR4, membrane receptors targeted by at-HMGB1, prevent the development of CIPN caused by paclitaxel (Figure 2A and Table 1) [9]. On the other hand, the role of TLR4 in the paclitaxel-induced CIPN appears to be different among species or strains. TAK-242, a TLR4 antagonist capable of penetrating into the CNS, prevents CIPN after paclitaxel treatment in C57BL/6 mice and Sprague–Dawley rats, but not in ddY mice (Table 1) [9,94]. It is to note that intraperitoneal (i.p.) administration of LPS-RS, a peripherally preferential TLR4 antagonist, does not prevent the CIPN in C57BL/6 mice (Table 1) [9], while intrathecal administration of LPS-RS blocks rat CIPN caused by paclitaxel (Table 1) [95]. Therefore, the CIPN caused by paclitaxel appears to involve central, but not peripheral, TLR4 in rats and C57BL/6 mice. Interestingly, there is a report showing that macrophage TLR9, an intracellular compartment receptor for HMGB1, participates in the CIPN caused by paclitaxel in male, but not female, mice, suggesting the existence of sex dimorphism in the role of TLR9 in the development of CIPN [96]. The same study has shown the involvement of TLR9 in the paclitaxel-induced release of TNF and CXCL1 from male, but not female, macrophages [96]. Similar sex dimorphisms have also been described in the role of TLR4 in pain processing [80,97]. Collectively, activation of RAGE and CXCR4 possibly by macrophage-derived at-HMGB1 may play a major part in CIPN due to paclitaxel.

### 3.3. Role of HMGB1 and Its Upstream/Downstream Molecules in CIPN Caused by Oxaliplatin

We have shown that the inactivation of HMGB1 with HMGB1-nAb or TMα and pharmacological blockade of RAGE, CXCR4, or TLR4 prevent CIPN in rodents treated with oxaliplatin (Figure 2B and Table 1) [10]. Peripheral TLR4 appears to be involved in the CIPN caused by oxaliplatin, because systemic administration of LPS-RS as well as TAK-242 exhibited a preventive effect in this CIPN model (Figure 2B and Table 1). Most surprisingly, macrophages do not play a major role in the CIPN caused by oxaliplatin, since depletion of macrophages by liposomal clodronate as well as inhibition of macrophage-derived HMGB1 by minocycline or ethyl pyruvate had no effect on the CIPN in mice treated with oxaliplatin [10]. An independent group [98] has suggested the involvement of macrophages in the same CIPN model, being apparently inconsistent with our results [10]. However, their study showed that liposomal clodronate only slightly (by 30%) reduced the oxaliplatin-induced allodynia, suggesting that the participation of macrophage-derived HMGB1 in the CIPN caused by oxaliplatin should be very minor, if any [99]. This notion is further supported by our findings that the number of macrophages in the sciatic nerve did not increase in mice treated with oxaliplatin. Thus, the origin of HMGB1 involved in CIPN caused by oxaliplatin is still open to question (Figure 2B). Alternatively, it is likely that HMGB1 derived from multiple cells including macrophages contributes to the development of CIPN following oxaliplatin treatment (Figure 2B), because oxaliplatin at relatively high concentrations (3–10 µM) causes HMGB1 release from Schwann cells and macrophage-like RAW264.7 cells in vitro [10]. Our study to explore other origins that secrete HMGB1 in response to oxaliplatin is now in progress.

## 4. Inactivation of HMGB1 for Prevention of CIPN

### 4.1. CIPN Prevention by Neutralization of HMGB1 with Monoclonal Antibodies

Many studies from different groups, including ours, have shown that HMGB1-nAb, developed by Nishibori et al. (Table 1) [21,22], strongly suppresses inflammatory pain [28], neuropathic pain by surgical injury of the sciatic nerve [31,91] or spinal nerve [76], and visceral pain following cystitis or pancreatitis [19,20,30,32]. The same HMGB1-nAb completely prevents the development of CIPN in rodents treated with paclitaxel, oxaliplatin, or vincristine (Table 1) [8,9,10]. The use of HMGB1-nAb is thus considered a promising approach to prevent CIPN, although it should be humanized before the clinical application.

### 4.2. Thrombin-Dependent Suppression of CIPN by Endogenous and Exogenous TM

TM, an endothelial membrane protein, consists of five domains: D1 (lectin-like domain), D2 (EGF-like domain), D3 (*O*-glycosylated serine-threonine-rich domain), D4 (transmembrane domain), and D5 (cytoplasmic domain). Thrombin binding to the D2 proteolytically converts protein C and TAFI into activated protein C (APC) and the activated form of TAFI (TAFIa), respectively (Figure 1D) [100]. TMα, consisting of three extracellular regions of TM (D1, D2, and D3), is generated by the protein expression system using Chinese hamster ovary (CHO) cells, and preserves the efficacy of TM in accelerating the thrombin-dependent activation of protein C and TAFI (Figure 1D) [11,35,36]. TMα as well as TM sequesters HMGB1 at the D1 and accelerates its degradation by thrombin binding to the D2 (Figure 1D and Table 1). Therefore, TMα inhibits the mechanical allodynia following intraplantar injection of at-HMGB1 or ds-HMGB1 in an endogenous thrombin-dependent manner [11,18,89]. TMα also dramatically reduces the endogenous HMGB1-dependent inflammatory hyperalgesia following intraplantar LPS [28], cystitis-related bladder pain [19,30], and pancreatitis-related pain [20,32]. Similarly, TMα prevents the development of CIPN in rodents treated with paclitaxel, vincristine, or oxaliplatin (Figure 2 and Table 1) [8,9,10]. A placebo-controlled, randomized, double-blind phase IIa study has confirmed the clinical usefulness of TMα in preventing CIPN in patients with colorectal cancer undergoing oxaliplatin-based chemotherapy (Table 1) [37]. It is important to note that patients undergoing anticoagulant therapy should be excluded in future clinical trials for evaluation of the anti-CIPN effect of TMα, considering our preclinical study showing that anti-coagulants cancelled the preventive effect of TMα on CIPN in mice (Figure 2B) [10,11]. Furthermore, our study has demonstrated that repeated administration of different anticoagulants aggravates CIPN and increases plasma HMGB1 levels in mice treated with a sub-effective dose of oxaliplatin [10], suggesting that the endothelial TM/thrombin system functions to reduce CIPN by degrading excessive HMGB1 released in response to oxaliplatin (Figure 2B) [11]. It has also been reported that the anti-CIPN effect of TMα in mice treated with oxaliplatin involves the thrombin-dependent production of APC and TAFIa, in addition to degradation of HMGB1, by TMα (Figure 2B and Table 1) [101]. The molecular mechanisms by which APC and TAFIa reduce the CIPN remain to be clarified. Apart from CIPN, TMα also reduces HMGB1-dependent symptoms in acute cystitis and pancreatitis accompanied by visceral pain [30,32] as well as disseminated intravascular coagulation (DIC) [102], and our ongoing studies focus on the effects of anticoagulants on HMGB1 levels in the blood stream in order to clarify the role of HMGB1 degradation by the endogenous TM/thrombin axis in those pathological conditions.

### 4.3. Other Candidates that Directly Inactivate HMGB1

Glycyrrhizin (glycyrrhizic acid), a component of licorice root, possesses anti-inflammatory and antiviral activities [103]. Direct binding of glycyrrhizin to both box A and box B of HMGB1 (*Kd* = ~150 µM) has been demonstrated by nuclear magnetic resonance (NMR) and fluorescence studies (Table 1), and the IC_50_ of glycyrrhizin is 50 µM in inhibiting cell migration of 3T3 fibroblasts stimulated with HMGB1 at 1 nM [104]. It has yet to be tested whether glycyrrhizin can prevent CIPN, although it inhibits diabetic neuropathy and retinopathy [92,105], dermatitis [106], chemotherapy and radiation resistance [107,108], brain injury by ischemic stroke [109,110], etc.

Methotrexate, a folic acid antagonist, is used in chemotherapy of tumors and autoimmune diseases including rheumatoid arthritis. Direct binding of methotrexate to two independent sites of HMGB1 has been demonstrated by surface plasmon resonance (SPR) analysis and electrophoretic mobility shift assay (EMSA) (Table 1) [111]. Methotrexate appears to inhibit the interaction of HMGB1 to RAGE, but not TLR4 [111]. It would be interesting to investigate the effect of methotrexate on CIPN.

Metformin, a biguanide derivative, is the first-line drug in the treatment of type 2 diabetes, and has also an anti-inflammatory activity. There is evidence that metformin directly binds to the C-terminal acidic tail of HMGB1, as demonstrated by a pull-down assay using full-length and C-terminal acidic tail-lacking HMGB1 (Table 1), an effect contributing to its anti-inflammatory effects [112]. Metformin inhibits the high glucose-induced upregulation of RAGE and HMGB1 in rat ventricular myocytes [113] and LPS-induced HMGB1 secretion in rabbit annulus fibrosus stem cells [114]. Most interestingly, preclinical studies have shown that metformin prevents CIPN in mice and rats treated with cisplatin and oxaliplatin, respectively (Table 1) [115,116].

(−)-Epigalocatechin-3-gllate (EGCG), a major effective component of green tea, is associated with many health benefits against multiple inflammatory diseases including rheumatoid arthritis [117]. EGCG is internalized into HMGB1-containing LC3-positive cytoplasmic vesicles (likely autophagosomes) in macrophages stimulated with LPS, leading to HMGB1 aggregation and inhibition of upregulation and extracellular release of HMGB1 [118]. A computational modeling study has shown that EGCG firmly binds to a region around C106 of HMGB1, leading to aggregation of HMGB1 (Table 1) [119]. Salicylic acid, a deacetylated form of aspirin, binds to the HMG-box domains of HMGB1, as assessed by NMR spectroscopic analysis (Table 1), and suppresses the chemoattractant activity of at-HMGB1 and the upregulation of proinflammatory cytokines and COX-2 induced by ds-HMGB1 [120]. The effects of EGCG and salicylic acid on CIPN have yet to be tested.

## 5. Blocking Membrane Receptors of HMGB1 for Prevention of CIPN

### 5.1. RAGE Antagonists

FPS-ZM1 was developed as a high-affinity RAGE-specific blocker through screening of 5000 compounds (Table 1) [67,121]. In a cell-free assay, FPS-ZM1 blocks binding of Aβ (*Ki* = 25 nM), S100B (*Ki* = 230 nM), and HMGB1 (*Ki* = 148 nM) [121] to immobilized recombinant soluble RAGE. FPS-ZM1 readily crosses the blood-brain barrier (BBB) and normalizes cognitive performance and cerebral blood flow responses in a mouse model of Alzheimer′s disease, aged *APP^SW/0^* mice [121]. FPS-ZM1 suppresses the endogenous HMGB1-dependent pancreatic [32] and bladder [19] pain. FPS-ZM1 also prevents the development of CIPN in mice treated with paclitaxel [9] or oxaliplatin [10] (Table 1).

Low molecular weight heparin (LMWH, parnaparin, MW: 4500~6500), an anticoagulant that preferentially inhibits factor Xa rather than factor IIa (thrombin), binds to RAGE at *Kd* value of 17 nM, as determined by SPR assay [122], although LMWH also has some sensitivity to HMGB1 itself [123]. LMWH strongly inhibits the mechanical allodynia following intraplantar administration of at-HMGB1 capable of stimulating RAGE, but not of ds-HMGB1 capable of stimulating TLR4, suggesting a possible contribution of RAGE blockade, but not HMGB1 inactivation, in the anti-allodynic effect of LMWH [16]. LMWH also prevents endogenous HMGB1-dependent pain, including cystitis-related bladder pain [30] and neuropathic pain, caused by surgical injury of the spinal nerve [76]. As does FPS-ZM1, LMWH prevents the development of CIPN in rodents treated with paclitaxel [9] or oxaliplatin [10] (Table 1).

Azeliragon (also called PF-04494700 or TTP488), an orally bioavailable small molecule antagonist of RAGE that can penetrate BBB, is now being evaluated for efficacy and safety in patients with Alzheimer’s disease, because stimulation of RAGE by amyloid β is involved in neurodegeneration (Table 1) [124,125,126]. Azeliragon blocks the interaction of RAGE with amyloid β, S100B, or HMGB1, as determined by a fluorescent polarization assay [67,124,125,126]. Given plenty of clinical evidence for the safety, azeliragon is one of the most promising candidates for an anti-CIPN agent, although neither preclinical nor clinical evidence for the effect of azeliragon on pain is available.

### 5.2. TLR Antagonists

Lipopolysaccharide of *Rhodobacter sphaeroides* (LPS-RS), one of the best-known TLR4 antagonists [127] (Table 1), suppresses the LPS-induced inflammatory responses both in vitro [128] and in vivo [129]. Chemical modification of the lipid A, a membrane-anchoring moiety of LPS, is well-established as an approach to develop TLR4-sensitive compounds [130]. Eritoran (E5564), a synthetic analogue of the lipid A, also antagonizes TLR4 (Table 1) [127] and suppresses the biological effects of LPS [131]. Both LPS-RS and eritoran antagonize TLR4 by targeting MD-2, a co-receptor of TLR4 [127]. FP7, a synthetic monosaccharide lipid A mimetic, also selectively blocks TLR4 signaling by binding to MD-2 and CD14 (Table 1) [132,133]. TAK-242 (also called CLI-095), a BBB-permeable small molecule, blocks TLR4 through covalent binding to Cys747 in the intercellular domain of TLR4 (Table 1) [134], thereby exhibiting anti-inflammatory activity [135]. Preclinical studies have shown the anti-CIPN effect of TAK-242 and LPS-RS in some animal models for CIPN (Table 1) [9,10,94,95]. TAK-242 is considered one of the best candidates for clinical application or as a seed in drug development, considering the chemical structure.

### 5.3. CXCR4 Antagonists

AMD3100 (also known as plerixafor), a small molecule, selectively blocks CXCR4 (Table 1) [136]. AMD3100 inhibits the mechanical allodynia caused by intrathecal administration of CXCL12, a ligand of CXCR4 [85], but not by intraplantar administration of HMGB1 alone [16]. However, AMD3100 prevents endogenous HMGB1-dependent pathological pain [20,32]. These reports are consistent with the evidence that HMGB1 in collaboration with CXCL12, not alone, stimulates CXCR4 (Figure 1C) [86,87]. Most interestingly, AMD3100 prevents the development of CIPN in mice treated with paclitaxel or oxaliplatin (Table 1) [9,10]. It is likely that endogenous HMGB1 and CXCL12 synergistically activate CXCR4, contributing to CIPN development. AMD3100 has been submitted to clinical trials for evaluation of its safety and efficacy in various cancer patients (clinical trial ID: NCT0128857, NCT02221479, NCT01696461, etc.), and is considered more suitable for clinical application as an anti-CIPN medicine than peptidic CXCR4 antagonists, such as BL-8040 (T140), LY2510924, and POL6326 (balixafortide).

## 6. Targeting Macrophages for Prevention of CIPN Due to Paclitaxel

As described above, ROS-dependent release of HMGB1 from macrophages plays a critical role in the development of CIPN in mice treated with paclitaxel [9], but not oxaliplatin [10]. Ethyl pyruvate, known to inhibit HMGB1 release from macrophages [137]; minocycline, an inhibitor of activation of macrophage/microglia [138]; or NAC, an antioxidant, prevents CIPN caused by paclitaxel in rodents [9] (Table 1), and may be worth evaluating its efficacy for humans.

**Table 1:** Drug candidates targeting HMGB1 or its upstream/downstream molecules for prevention of CIPN.

Chemicals (Alternative Name)	Structure	Mechanisms of Action	Ref.	Prevention of CIPN (Administration Route; Animal; Anticancer Drug)	Ref.
HMGB1-nAb	IgG (MW 150,000)	Binding to and inactivation of HMGB1	[21,22]	Yes (i.p.; rat; PCT, VCR) Yes (i.p.; mouse; PCT) Yes (i.p.; mouse; OHP)	[8] [9] [10]
TMα	Protein (MW 64,000)	Binding to HMGB1 and promotion of degradation of HMGB1 by thrombin	[18,35,36,89]	Yes (i.p.; rat; PCT, VCR) Yes (i.p.; mouse; PCT) Yes (i.p., mouse; OHP) Yes (i.v.; rat; OHP) Yes (i.v.; human; OHP)	[8] [9] [10] [101] [37]
Glycyrrhizin (glycyrrhizic acid)	Small molecule (MW 822.9)	Binding to both box A and box B of HMGB1	[104]	N.D.	
Methotrexate	Small molecule (MW 454.44)	Binding to HMGB1 and inhibition of HMGB1/RAGE interaction	[111]	N.D.	
Metformin	Small molecule (MW 129.16)	Binding to the C-terminal acidic tail of HMGB1	[112]	Yes (i.p.; rat; OHP) Yes (i.p.; mouse; CDDP)	[116] [115]
EGCG	A green tea component, small molecule (MW 458.37)	Binding to HMGB1 and induction of aggregation of HMGB1	[119]	N.D.	
Salicylic acid	Aspirin metabolite, small molecule (MW 138.12)	Bonding to box A and box B of HMGB1	[120]	N.D.	
FPS-ZM1	Small molecule (MW 327.8)	Blockade of RAGE	[67,121]	Yes (i.p.; mouse; PCT) Yes (i.p.; mouse; OHP)	[9] [10]
LMWH (parnaparin)	(MW 4500~6500)	Blockade of RAGE	[122,123]	Yes (i.p.; mouse; PCT) Yes (i.p.; mouse; OHP)	[9] [10]
Azeliragon (PF-04494700 or TTP488)	Small molecule (MW 532.1)	Blockade of RAGE	[67,124,125,126]	N.D.	
LPS-RS	LPS from the photosynthetic bacterium *Rhodobacter sphaeroides*	Blockade of TLR4	[127,139]	Yes (i.t.; rat; PCT) No (i.p.; mouse; PCT) Yes (i.p.; mouse; OHP)	[95] [9] [10]
Eritoran (E5564)	Synthetic lipid A analogue (MW 1313.7)	Blockade of TLR4	[127,131]	N.D.	
FP7	Synthetic monosaccharide lipid A mimetic with about half MW of eritoran	Blockade of TLR4	[132,133]	N.D.	
TAK-242 (CLI-095)	Small molecule (MW 361.82)	Blockade of TLR4	[134,135]	Yes (i.v.; rat; PCT) Yes (i.p.; mouse *; PCT) No (i.p.; mouse **; PCT) Yes (i.p.; mouse, OHP)	[94] [9] [9] [10]
AMD3100 (plerixafor)	Small bicyclam molecule (MW 129.16)	Blockade of CXCR4	[136]	Yes (i.p.; mouse; PCT) Yes (i.p.; mouse; OHP)	[9] [10]
Ethyl pyruvate	Stable lipophilic pyruvate derivative, small molecule (MW 116.11)	Inhibition of HMGB1 secretion from macrophage	[137]	Yes (i.p.; mouse; PCT) No (i.p.; mouse; OHP)	[9] [10]
Minocycline	Small molecule (MW 457.48)	Inhibition of macrophage/microglia	[138]	Yes (i.p.; mouse; PCT) No (i.p.; mouse; OHP)	[9] [10]
NAC	Small molecule (MW 163.20)	Anti-oxidation, suppression of HMGB1 release	[9,55]	Yes (i.p.; mouse; PCT)	[9]

Ref., References; *, C57BL/6 strain; **, ddY strain; N.D., not determined; PCT, paclitaxel; VCR, vincristine; OHP, oxaliplatin; CDDP, cisplatin; HMGB1-nAb, anti-HMGB1-neutralizing antibody; EGCG, (-)-epigalocatechin-3-gallate; LMWH, low molecular weight heparin; LPS-RS, lipopolysaccharide from *Rhodobacter sphaeroides*; NAC, *N*-acetylcysteine.

## 7. Conclusions

HMGB1 is now considered a key molecule responsible for the development of pathological pain including CIPN. HMGB1 and its upstream/downstream molecules are thus considered promising targets to develop agents to prevent CIPN. In particular, the clinical effectiveness of TMα, capable of inactivating HMGB1, in preventing the CIPN due to oxaliplatin in humans [37] is encouraging, because many compounds targeting other molecules, which were effective in preclinical studies using animal models for CIPN, did not show significant efficacy or enough safety in clinical trials [1].

## Data Availability

Not applicable.
